# Expression of Concern for Correlation Between Sun Protection Factor and Antioxidant Activity, Phenol and Flavonoid Contents of Some Medicinal Plants [Iran J Pharm Res. 2014;13(3): e125511]

**DOI:** 10.5812/ijpr-157931

**Published:** 2024-11-10

**Authors:** Editor-in-Chief IJPR

**Affiliations:** 1 School of Pharmacy, Shahid Beheshti University of Medical Sciences, Tehran, Iran

The editorial board of the IJ Pharmaceutical Research wishes to issue an Expression of Concern regarding the article titled "Correlation between Sun Protection Factor and Antioxidant Activity, Phenol and Flavonoid Contents of some Medicinal Plants" (1).

Concerns have been raised about several aspects of the article, including:

- The claim of a "good correlation" between Sun Protection Factor (SPF) and phenolic content, while the reported correlation coefficient of 0.55 suggests only a moderate correlation, which may overstate the results.

- The very high SPF values reported for some extracts, such as an SPF of 24.47 for the ultrasonic extract of *Crataegus pentagyna*, lack sufficient explanation or comparison to established sunscreen products, which warrants further discussion and validation.

- The recommendation to use these extracts "alone or as additives in other sunscreen formulations" is based solely on in vitro SPF measurements, without any in vivo testing or safety evaluation, making the recommendation premature.

- The absence of raw spectrophotometric data used to calculate SPF values limits the ability to independently verify the results.

- The study's sample size is limited to only four plant species, which restricts the strength and generalizability of the conclusions.

- There is limited discussion of the study's limitations or potential sources of error.

The journal is currently reviewing these concerns and is in the process of determining whether corrections or clarifications from the authors are necessary. Readers are advised to interpret the findings with caution until further notice.

Sincerely Yours, 

Editorial Board 

IJ Pharmaceutical Research
